# Multifactorial risk stratification for central lymph node metastasis in papillary thyroid carcinoma: a predictive model integrating clinical and tumor characteristics

**DOI:** 10.3389/fonc.2025.1586307

**Published:** 2025-05-21

**Authors:** Huaiyu Yang, Liyuan Wei, Jiaxin Qian, Wensheng Liu

**Affiliations:** Department of Head and Neck Surgical Oncology, National Cancer Center, National Clinical Research Center for Cancer, Cancer Hospital, Chinese Academy of Medical Sciences and Peking Union Medical College, Beijing, China

**Keywords:** papillary thyroid carcinoma, central lymph node metastasis, risk factor (RF), Nomogram model, retrospective study

## Abstract

**Background:**

With the increasing prevalence of papillary thyroid carcinoma PTC) and advancements in auxiliary examination technology, the holistic detection rate of malignant thyroid nodules, particularly small ones, continues to rise. However, there remains controversy surrounding the optimal treatment for PTC, and a crucial factor influencing treatment decisions is the status of central lymph node metastasis (CLNM). There is a lack of research on the relationship between clinical laboratory results and tumor characteristics observed during surgery and CLNM status. Therefore, our research aims to systematically explore the risk factor of CLNM in patients with PTC.

**Methods:**

We systematically gathered and analyzed clinical features and pathological data of 2,435 PTC patients who underwent surgery. After variable screening, the selected variables were included in logistic regression analysis, and a Nomogram prediction model was constructed according to the analysis results. To investigate the risk factors for CLNM in patients with PTC.

**Result:**

This study included a total of 2,435 patients diagnosed with PTC, among whom 933 patients were confirmed as CLNM by postoperative pathology. Univariate and multivariate regression analysis identified age, serum TRAb levels, calcification, multifocality, extrathyroidal invasion, tumor size, and tumor location as risk factors associated with CLNM. The prediction model based on these risk factors demonstrated robust accuracy with an AUC of 0.76. Clinical decision curve analysis indicated that aside from a small range of low threshold probabilities, intervening based on the model’s predictions can yield greater clinical benefit.

**Conclusion:**

Key risk factors for CLNM in PTC patients include young age, high serum thyrotropin receptor antibody (TR-Ab) levels, calcification, multifocality, extrathyroidal extension, larger tumor size, and tumor location in the middle or lower poles of the thyroid. The clinical prediction model established based on these critical risk factors can provide a more accurate reference standard for clinical decision-making in practice.

## 1 Introduction

Thyroid cancer, predominantly papillary thyroid carcinoma (PTC), constitutes the most prevalent endocrine malignancy globally, accounting for over 90% of cervical region neoplasms in head and neck oncology as defined by the World Health Organization (WHO) classification of endocrine tumors (1), with PTC being the most prevalent type of pathology. The gradually incremental incidence of thyroid malignancies, along with advancements in imaging techniques, has resulted in a higher detection rate of PTC, particularly in cases involving small foci (2). At present, surgical intervention remains the primary treatment modality for PTC (3). The prevailing strategy for managing regional lymph nodes involves preventive lymph node dissection (PLND), which facilitates the removal of both macroscopic and microscopic metastatic foci (4), thereby contributing to precise clinical staging. However, retrospective studies have shown that approximately 30–80% of PTC patients undergoing PLND have pathologically confirmed lymph node metastases (5). Furthermore, this procedure significantly increases the incidence of short-term and permanent postoperative complications, including hypocalcemia, recurrent laryngeal nerve paralysis, and transient parathyroid dysfunction (6, 7). Consequently, the preoperative identification of high-risk PTC patients with central lymph node metastasis (CLNM) is of paramount importance. This study systematically compiles prevalent clinical characteristics, examines risk factors associated with CLNM, develops a predictive model, and assesses its efficacy in determining CLNM risk. The objective of this model is to furnish clinicians with a meaningful tool to assess the CLNM status of PTC patients, thereby enhancing the precision of clinical decision.

## 2 Materials and methods

### 2.1 Patient population

**Figure 1:**
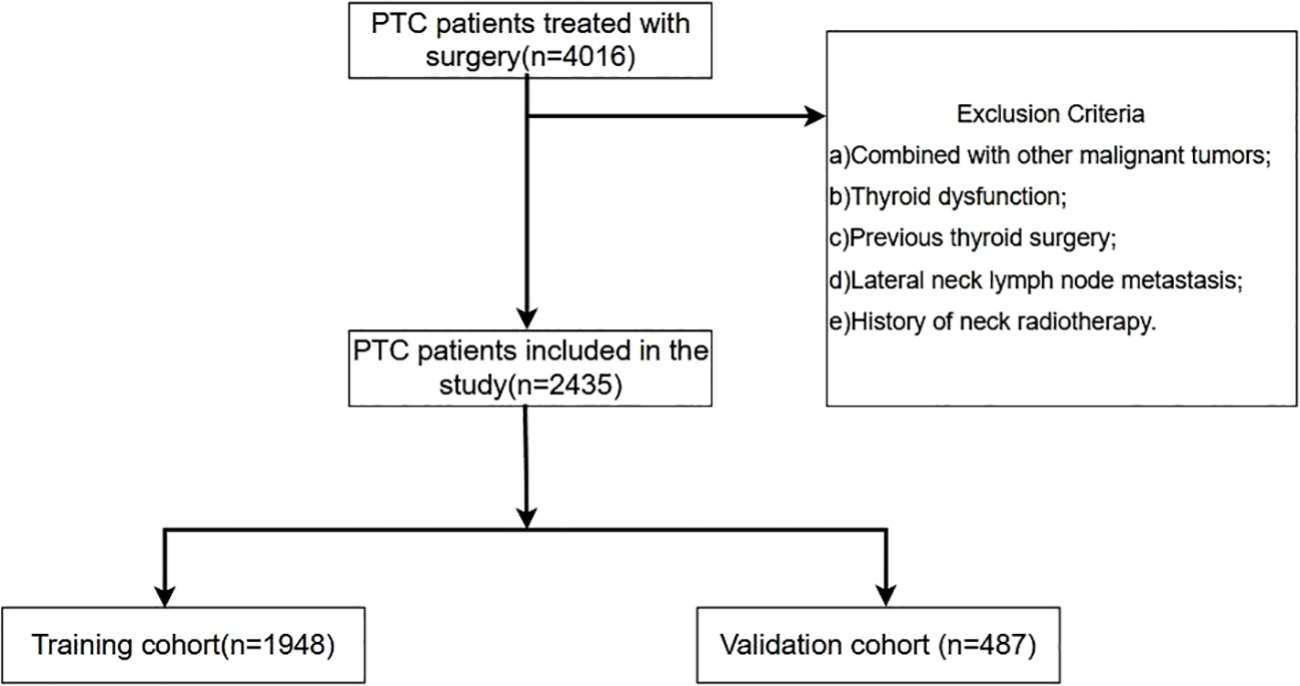
Schematic diagram of the procedure for inclusion and exclusion of patients in this study. A total of 4016 PTC patients were consecutively reviewed in this study. According to the prespecified inclusion and exclusion criteria, 2435 PTC patients were finally included in this study. According to the ratio of 8:2, they were randomly divided into training set (1948 cases) and validation set (487 cases).

This retrospective cohort study included patients diagnosed with PTC who underwent thyroid lobectomy or total thyroidectomy with PLND at the Department of Head and Neck Surgery, Cancer Hospital of the Chinese Academy of Medical Sciences (CICAMS), between January 1, 2015, and December 1, 2022.The inclusion criteria encompassed: a) patients with an initial diagnosis of PTC confirmed through preoperative, core needle biopsy pathology, aspiration cytology, and postoperative pathology; and b) patients who underwent thyroid lobectomy accompanied by PLND, with comprehensive preoperative ultrasound and thyroid function assessment, and postoperative pathological record. And the exclusion criteria encompassed the following: a) individuals with a prior history of neck radiotherapy or other malignancies; b) individuals exhibiting preoperative thyroid function test results indicative of hyperthyroidism or hypothyroidism; c) individuals with a history of partial thyroidectomy or other thyroid-related surgical procedures; d) individuals whose preoperative biopsy pathology revealed lateral neck lymph node metastasis. Finally, 2,435 patients satisfied the criteria, comprising 933 patients with CLNM, and 1,502 patients no-CLNM. Subsequently, Randomly divided the patients into training cohort (1,948) and validation cohort (487) following an 8:2 distribution ratio. The process for patient enrollment is shown in **Figure 1**. The experimental scheme was designed in accordance with the ethical principles of the Helsinki Declaration and authorized by the Ethics Committee of the Cancer Hospital, Chinese Academy of Medical Sciences.

### 2.2 Baseline data information

We systematically collected data from patients included in the study, encompassing basic demographic information, laboratory test results, thyroid ultrasound findings, surgical methods, surgical records, and postoperative pathological outcomes. The demographic data comprise age, BMI, and sex. Laboratory indicators include consequence of thyroid-stimulating hormone (TSH), thyroglobulin (Tg), thyrotropin receptor antibodies (TR-Ab), and thyroglobulin antibodies (TG-Ab) levels. The thyroid ultrasound findings provided a comprehensive assessment of primary tumor site, including the nodule localization and the number of thyroid nodules, calcification, extrathyroidal extension (ETE), and tumor diameter. Multifocality was characterized by the detection of two or more thyroid nodules via ultrasound, which were later pathologically confirmed as PTC (8). Tumor size was determined by measuring the maximum diameter of the nodule on ultrasound, with these measurements subsequently corroborated through pathological analysis.

To ensure the consistency of image quality, all preoperative ultrasound images were completed by the same grade of sonographer with reference to the Chinese version of Thyroid Imaging Reporting and Data System (C-TIRADS), and calibrated by a senior chief sonographer. In this study, the classification criteria for thyroid nodule location by sonographers and surgeons was based on the tripartition of the thyroid gland lobe. The description of thyroid nodule location in preoperative ultrasound reports and surgical records of PTC patients was reviewed completely, and the specific results were recorded for subsequent statistical analysis of this study. In the data analysis phase, we used Kappa coefficient to evaluate the inter-rater reliability of tumor localization between surgeons and radiologists. The results showed that the inter-rater reliability of tumor localization between surgeons and radiologists was 0.880 (p < 0.0001, 95%CI 0, 862 to 0.899). The Kappa coefficient was greater than 0.8, indicating excellent correlation between the two sets of data. The anatomical location of thyroid nodules, including the upper pole, middle pole, lower pole and isthmus, and the degree of extrathyroidal extension observed during the operation were recorded.

The complete collection of postoperative pathological information should include the following: the histopathological subtype of papillary thyroid carcinoma (classic variant, diffuse sclerosing variant, follicular variant, tall cell variant, etc.); whether the tumor exhibits multifocal growth and the specific number of tumor foci; the maximum diameter of the primary tumor (in millimeters); surrounding tissue invasion such as the thyroid capsule, trachea, or recurrent laryngeal nerve; the presence of coexisting chronic thyroiditis; and whether ipsilateral CLNM is present, along with the number of metastatic lymph nodes.

All enrolled patients’ surgical records were retrieved through the HIS system. After verifying the completeness of the surgical documentation, intraoperative findings from PTC patients meeting the inclusion criteria were collected. This included the lobe of the thyroid gland where the primary tumor was located (upper pole, middle third, lower pole, or isthmus), the presence of extrathyroidal extension (ETE), and the extent of such invasion.

### 2.3 Statistical analysis

All statistical analyses applied in our study were conducted utilizing R software (version 4.4.0), with statistical significance established at p-value less than 0.05. Categorical data were expressed as percentage, while continuous variables were reported as means ± SD or medians (interquartile range, 1 and 3) after the primary analysis, depending on the normality of the variables. Collinearity among variables was assessed using a correlation coefficient matrix, variance inflation factor (VIF), and tolerance. Variables were screened based on collinearity diagnostics and subsequently included in univariable logistic regression analysis to calculate odds ratios (OR). After screening, these variables were incorporated into a multivariable logistic analysis. And then utilizing these selected variables developed clinical prediction model for CLNM. The receiver operating characteristic curve (ROC) and the area under the curve(AUC), decision curve analysis (DCA) and calibration curve were adopted to verify the performance of the prediction model.

## 3 Results

### 3.1 Demographic and clinical profiles at enrollment

Our study gathered data from 2,435 patients with PTC, comprising1,502 patients without CLNM and 933 patients with CLNM. A summary of the clinical data characteristics for both groups is presented in **Table 1**.

**Table 1:** Clinical baseline characteristics of total patients cohort.

Variables	Total (n = 2435)	No-CLNM (n = 1502)	CLNM (n = 933)	*P*
Age	43.74 ± 10.09	45.47 ± 10.07	40.94 ± 9.48	**<.001**
Weight	67.12 ± 12.94	67.43 ± 13.15	66.62 ± 12.60	0.133
Height	164.17 ± 13.87	163.58 ± 10.50	165.12 ± 17.97	**0.008**
Diameter (ultrasound)	8.62 ± 4.51	7.50 ± 3.73	10.42 ± 5.05	**<.001**
Diameter (pathology)	7.34 ± 4.11	6.32 ± 3.51	8.98 ± 4.46	**<.001**
Tg	45.29 ± 129.06	43.88 ± 145.61	47.57 ± 96.28	0.555
TR-Ab	0.45 ± 0.37	0.42 ± 0.31	0.49 ± 0.46	**<.001**
TG-Ab	122.39 ± 408.36	126.58 ± 407.33	115.60 ± 410.24	0.578
BMI	31.50 ± 135.58	30.45 ± 122.09	33.18 ± 154.92	0.630
Sex, n(%)				**0.002**
female	1734 (71.21)	1104 (73.50)	630 (67.52)	
male	701 (28.79)	398 (26.50)	303 (32.48)	
Calcification, n(%)				**<.001**
No	1323 (54.33)	975 (64.91)	348 (37.30)	
Yes	1112 (45.67)	527 (35.09)	585 (62.70)	
Multifocal (ultrasound), n(%)				**<.001**
Multifocal	447 (18.36)	232 (15.45)	215 (23.04)	
Unifocal	1988 (81.64)	1270 (84.55)	718 (76.96)	
Diameter group-1, n(%)				**<.001**
>7mm	1275 (52.36)	606 (40.35)	669 (71.70)	
≤7mm	1160 (47.64)	896 (59.65)	264 (28.30)	
Diameter group-2, n(%)				**<.001**
>10mm	590 (24.23)	236 (15.71)	354 (37.94)	
≤10mm	1845 (75.77)	1266 (84.29)	579 (62.06)	
ETE (ultrasound), n(%)				**<.001**
Negative	2303 (94.58)	1379 (91.81)	924 (99.04)	
Positive	132 (5.42)	123 (8.19)	9 (0.96)	
Bilateral/Unilateral, n(%)				**<.001**
Bilateral	575 (23.61)	443 (29.49)	132 (14.15)	
Unilateral	1860 (76.39)	1059 (70.51)	801 (85.85)	
Lobes, n(%)				**<.001**
Bilateral	575 (23.61)	443 (29.49)	132 (14.15)	
Isthmus	24 (0.99)	20 (1.33)	4 (0.43)	
Lift	846 (34.74)	493 (32.82)	353 (37.83)	
Right	990 (40.66)	546 (36.35)	444 (47.59)	
Location, n(%)				0.156
Lower	436 (19.34)	254 (18.53)	182 (20.61)	
Middle	1228 (54.48)	769 (56.09)	459 (51.98)	
Upper	590 (26.18)	348 (25.38)	242 (27.41)	
Age group-1, n(%)				**<.001**
≤40	830 (34.09)	403 (26.83)	427 (45.77)	
>40	1605 (65.91)	1099 (73.17)	506 (54.23)	
Age group-2, n(%)				**<.001**
≤45	1335 (54.83)	708 (47.14)	627 (67.20)	
>45	1100 (45.17)	794 (52.86)	306 (32.80)	
Multifocal (pathology), n(%)				**<.001**
Unifocal	1850 (75.98)	1222 (81.36)	628 (67.31)	
Multifocal	585 (24.02)	280 (18.64)	305 (32.69)	
Number of nodules, n(%)				**<.001**
1	1856 (76.22)	1228 (81.76)	628 (67.31)	
2	364 (14.95)	183 (12.18)	181 (19.40)	
3	215 (8.83)	91 (6.06)	124 (13.29)	
ETE (pathology), n(%)				**<.001**
Negative	1053 (43.33)	745 (49.60)	308 (33.19)	
Capsular	324 (13.33)	203 (13.52)	121 (13.04)	
Extracapsular	1053 (43.33)	554 (36.88)	499 (53.77)	

The meaning of the bold values indicated that there were clear statistical differences in the variable in different central lymph node metastasis status groups of papillary thyroid carcinoma (p < 0.05).

We used the `createDataPartition()` function in the R package `caret` to randomly divide the total dataset into a training set (1,948 cases) and a validation set (487 cases). Based on the outcome variable (lymph node metastasis status) as the basis for stratification, the proportion parameter for the training set was set to p = 0.8. The random seed was fixed at 1234 to ensure the repeatability of the results. In the training cohort, there were 743 patients diagnosed with CLNM and 1,205 patients without CLNM. Similarly, in the validation cohort, 190 patients were identified as having CLNM and 297 patients were identified as having no CLNM. After dividing the training set and the validation set, the balance analysis of the data between the training set and the validation set was carried out. The results showed that, Apart from the number of tumor foci identified through ultrasound and subsequently confirmed by postoperative pathology, There were no significant differences in other clinical and pathological features between the two groups. Summary of the clinical data characteristics for both groups is presented in **Table 2**.

**Table 2:** Baseline characteristics of the patients of training and validation cohort.

Variables	Total (n = 2435)	Validation cohort (n = 487)	Training cohort (n = 1948)	*P*
Age	43.74 ± 10.09	43.85 ± 9.83	43.71 ± 10.16	0.773
Weight	67.12 ± 12.94	67.27 ± 12.81	67.09 ± 12.98	0.777
Height	164.17 ± 13.87	164.07 ± 12.63	164.20 ± 14.16	0.860
Diameter(ultrasound)	8.62 ± 4.51	8.61 ± 4.50	8.62 ± 4.52	0.986
Diameter(pathology)	7.34 ± 4.11	7.39 ± 4.29	7.33 ± 4.06	0.745
Tg	45.29 ± 129.06	45.32 ± 125.28	45.28 ± 130.11	0.995
TR-Ab	0.45 ± 0.37	0.45 ± 0.39	0.45 ± 0.37	0.893
TG-Ab	122.39 ± 408.36	104.87 ± 312.39	127.08 ± 430.39	0.345
BMI	31.50 ± 135.58	35.93 ± 174.86	30.39 ± 123.83	0.420
Sex, n(%)				0.840
female	1734 (71.21)	345 (70.84)	1389 (71.30)	
male	701 (28.79)	142 (29.16)	559 (28.70)	
Age group-1, n(%)				0.831
≤40	830 (34.09)	164 (33.68)	666 (34.19)	
>40	1605 (65.91)	323 (66.32)	1282 (65.81)	
Age group-2, n(%)				1.000
≤45	1335 (54.83)	267 (54.83)	1068 (54.83)	
>45	1100 (45.17)	220 (45.17)	880 (45.17)	
Calcification, n(%)				0.887
No	1323 (54.33)	266 (54.62)	1057 (54.26)	
Yes	1112 (45.67)	221 (45.38)	891 (45.74)	
Multifocal(ultrasound), n(%)				**0.030**
Unifocal	1988 (81.64)	381 (78.23)	1607 (82.49)	
Multifocal	447 (18.36)	106 (21.77)	341 (17.51)	
Diameter group-1, n(%)				0.224
≤7mm	1160 (47.64)	244 (50.10)	916 (47.02)	
>7mm	1275 (52.36)	243 (49.90)	1032 (52.98)	
Diameter group-2, n(%)				0.723
≤10mm	1845 (75.77)	366 (75.15)	1479 (75.92)	
>10mm	590 (24.23)	121 (24.85)	469 (24.08)	
ETE(ultrasound), n(%)				0.325
Negative	2303 (94.58)	465 (95.48)	1838 (94.35)	
Positive	132 (5.42)	22 (4.52)	110 (5.65)	
Bilateral/Unilateral, n(%)				0.340
Unilateral	1860 (76.39)	364 (74.74)	1496 (76.80)	
Bilateral	575 (23.61)	123 (25.26)	452 (23.20)	
Lobes, n(%)				0.798
Lift	846 (34.74)	163 (33.47)	683 (35.06)	
Right	990 (40.66)	196 (40.25)	794 (40.76)	
Isthmus	24 (0.99)	5 (1.03)	19 (0.98)	
Bilateral	575 (23.61)	123 (25.26)	452 (23.20)	
Location, n(%)				0.063
Upper	436 (19.34)	73 (15.94)	363 (20.21)	
Medium	1228 (54.48)	251 (54.80)	977 (54.40)	
Lower	590 (26.18)	134 (29.26)	456 (25.39)	
Multifocal(pathology), n(%)				**0.033**
Unifocal	1850 (75.98)	352 (72.28)	1498 (76.90)	
Multifocal	585 (24.02)	135 (27.72)	450 (23.10)	
Number of nodules, n(%)				0.053
1	1856 (76.22)	351 (72.07)	1505 (77.26)	
2	364 (14.95)	87 (17.86)	277 (14.22)	
3	215 (8.83)	49 (10.06)	166 (8.52)	
ETE (pathology), n(%)				0.710
Negative	1053 (43.33)	217 (44.56)	836 (43.03)	
Capsular	324 (13.33)	60 (12.32)	264 (13.59)	
Extracapsular	1053 (43.33)	210 (43.12)	843 (43.39)	

The meaning of the bold values indicated that there were clear statistical differences in the variable in different central lymph node metastasis status groups of papillary thyroid carcinoma (p < 0.05).

### 3.2 Relation between clinicopathological features of PTC and CLNM in all patient cohort

In this study, CLNM was identified in 933 patients (38.30%) with PTC. A comprehensive pre-analysis of all patients revealed a noteworthy association between CLNM and multiple factors, including age (p < 0.01), maximum tumor diameter (p < 0.01),serum TR-Ab levels (p < 0.01), sex (p < 0.01), calcification (p < 0.01), multifocality (p < 0.01), the number of tumor foci (p < 0.01) and extrathyroidal invasion (p < 0.01) observed via ultrasound, tumor location (p < 0.01), and ETE (p < 0.01). correspondingly, no significant correlations were observed regarding weight, BMI, serum triglyceride (Tg) levels, serum TG-antibody (TG-Ab) levels, or the laterality of the tumor (p > 0.05).

Building on previous studies (9), we classified the maximum tumor diameter using thresholds of >7 mm and >10 mm. Significant differences in CLNM rates were observed between these groups. The >10 mm threshold (OR: 3.75, 95% CI: 3.14–4.47) exhibited superior accuracy and specificity than the >7 mm threshold. Detailed results are presented in **Table 3**.

**Table 3:** Univariate logistic regression analysis of total patients cohort.

Variables	*P*	OR (95%CI)
Age	**<.001**	0.95 (0.95 ~ 0.96)
Weight	0.134	1.00 (0.99 ~ 1.00)
Height	**0.015**	1.01 (1.01 ~ 1.02)
Diameter(ultrasound	**<.001**	1.17 (1.15 ~ 1.20)
Diameter(pathology)	**<.001**	1.18 (1.16 ~ 1.21)
Tg	0.556	1.00 (1.00 ~ 1.00)
TR-Ab	**<.001**	1.57 (1.24 ~ 1.99)
TG-Ab	0.579	1.00 (1.00 ~ 1.00)
BMI	0.632	1.00 (1.00 ~ 1.00)
Sex		
female		1.00 (Reference)
male	**0.002**	1.33 (1.12 ~ 1.60)
Calcification		
No		1.00 (Reference)
Yes	**<.001**	3.11 (2.62 ~ 3.69)
Multifocal		
Unifocal		1.00 (Reference)
Multifocal	**<.001**	1.64 (1.33 ~ 2.02)
Number of nodules (ultrasound)		
1		1.00 (Reference)
≥2	**<.001**	0.42 (0.31 ~ 0.56)
Diameter group-1		
≤7mm		1.00 (Reference)
>7mm	**<.001**	3.28 (2.71 ~ 3.97)
Diameter group-2		
≤10mm		1.00 (Reference)
>10mm	**<.001**	3.75 (3.14 ~ 4.47)
ETE (ultrasound)		
Negative		1.00 (Reference)
Positive	**<.001**	3.45 (2.38 ~ 5.00)
Bilateral/Unilateral		
Unilateral		1.00 (Reference)
Bilateral	0.072	0.84 (0.69 ~ 1.02)
Thyroid gland lobe		
Lift		1.00 (Reference)
Right	0.929	0.99 (0.82 ~ 1.20)
Isthmus	0.156	0.51 (0.20 ~ 1.29)
Bilateral	0.088	0.83 (0.66 ~ 1.03)
Location		
Upper		1.00 (Reference)
Medium	**<.001**	1.64 (1.33 ~ 2.02)
Lower	**0.010**	1.41 (1.09 ~ 1.83)
ETE-1(intraoperative)		
Negative		1.00 (Reference)
Capsular	**<.001**	1.59 (1.27 ~ 1.99)
Extracapsular	**<.001**	2.32 (1.74 ~ 3.09)
ETE-2(intraoperative)		
Negative		1.00 (Reference)
Positive	**<.001**	1.82 (1.51 ~ 2.19)
Multifocal(pathology)		
Unifocal		1.00 (Reference)
Multifocal	**<.001**	2.12 (1.76 ~ 2.56)
Number of nodules (pathology)		
1		1.00 (Reference)
2	**<.001**	1.93 (1.54 ~ 2.43)
≥3	**<.001**	2.66 (2.00 ~ 3.55)
ETE (pathology)		
Negative		1.00 (Reference)
Capsular	**0.006**	1.44 (1.11 ~ 1.87)
Extracapsular	**<.001**	2.18 (1.82 ~ 2.61)

The meaning of the bold values indicated that there were clear statistical differences in the variable in different central lymph node metastasis status groups of papillary thyroid carcinoma (p < 0.05).

### 3.3 Univariate logistic regression analysis of clinicopathological predictors for CLNM in a training cohort

Based on the comprehensive statistical analysis of the full cohort of participants, the correlation coefficient matrix (correlation coefficient value less than 0.8. **Table 4**), variance inflation factor (VIF value less than 5) and tolerance were used to diagnose the collinearity between the variables (**Table 5**). Combined with clinical experience, the collinearity factor was removed from the training using principal component analysis. Variables with p < 0.05 were included in subsequent analyses. The findings revealed that, within the training cohort, the following were identified as potential risk factors for CLNM: male (OR: 1.33, 95% CI: 1.12–1.60), calcification (OR: 3.11, 95% CI: 2.62–3.69), multifocality (OR: 1.64, 95% CI: 1.33–2.02), ETE (ultrasound) (OR: 3.45, 95% CI: 2.38–5.00), and tumor location in the mid-thyroid (OR: 1.41, 95% CI: 1.09–1.83),lower pole (OR: 1.64, 95% CI: 1.33–2.02), ET(intraoperative) (OR: 1.82, 95% CI: 1.51–2.19), maximum tumor diameter (p < 0.001), high level serum TR-Ab (p < 0.01), and younger age (p < 0.01) were identified as potential risk factors of CLNM.

**Table 4:** Matrix table of correlation coefficients based on the full cohort of PTC patients.

Pearson correlation coefficients																		
	Status	Age	Weight	Height	Tg	TR-Ab	TG-Ab	BMI	Sex	Calcification	Multifocal	Diameter	Diametergroup-1	ETE	Unilateral/bilateral	Gland lobe	Location	ETE(intraoperative)
Status	1																	
Age	-0.232**	1																
Weight	-0.019	0.016	1															
Height	0.056*	-0.169**	0.422**	1														
Tg	0.010	0.006	0.011	-0.020	1													
TR-Ab	0.071**	-0.085**	-0.007	0.039	-0.015	1												
TG-Ab	-0.046	0.044	-0.066**	0.005	-0.053*	0.159**	1											
BMI	0.012	0.004	-0.007	-0.738**	-0.009	-0.026	-0.016	1										
Sex	0.087**	-0.127**	0.564**	0.507**	-0.018	0.022	-0.106**	-0.034	1									
Calcification	0.284**	-0.154**	0.014	0.074**	0.027	0.020	0.048*	-0.056*	0.073**	1								
Multifocal	-0.132**	0.027	-0.069**	-0.018	-0.045	-0.047	0.008	-0.050*	-0.045	-0.081**	1							
Diameter	0.303**	-0.111**	-0.019	0.062*	0.046	0.023	-0.015	-0.045	0.039	0.318**	-0.103**	1						
Diameter group-1	0.217**	-0.105**	0.017	0.073**	0.040	0.007	-0.019	-0.035	0.052*	0.213**	-0.119**	0.810**	1					
ETE	-0.190**	0.123**	0.073**	-0.005	0.038	0.037	-0.011	-0.012	0.022	-0.126**	0.071**	-0.323**	-0.139**	1				
Unilateral/bilateral	0.184**	-0.024	-0.021	0.026	0.001	-0.015	0.035	-0.041	0.012	0.262**	0.156**	0.534**	0.308**	-0.450**	1			
Gland lobe	0.175**	-0.039	-0.011	0.044	-0.011	-0.011	0.030	-0.043	0.017	0.232**	0.136**	0.486**	0.286**	-0.413**	0.919**	1		
Location	-0.021	-0.007	-0.032	-0.025	-0.027	-0.031	0.030	0.024	-0.001	-0.010	0.075**	0.063*	0.061*	-0.056*	0.067**	0.046	1	
ETE(intraoperative)	0.012	0.035	0.013	-0.014	-0.009	-0.029	-0.005	0.035	0.019	0.054*	-0.106**	-0.127**	-0.144**	0.070**	-0.012	0.014	-0.071**	1

* p<0.05 ** p<0.01.

**Table 5:** Collinearity diagnosis (VIF value and tolerance) based on data from all PTC patients.

Diagnostic results of collinearity		
Variables	VIF value	Tolerance
Age	1.208	0.828
Weight	1.817	0.550
Height	5.849	0.171
Tg	1.015	0.985
TR-Ab	1.051	0.952
TG-Ab	1.071	0.934
BMI	4.097	0.244
Sex	2.219	0.451
Calcification	1.222	0.818
Multifocal	1.163	0.860
Diameter	4.331	0.231
Diametergroup-1	3.219	0.311
ETE	1.387	0.721
Unilateral/bilateral	7.501	0.133
Gland lobe	6.549	0.153
Location	1.024	0.977
ETE (intraoperative)	1.072	0.933

**Figure 2:**
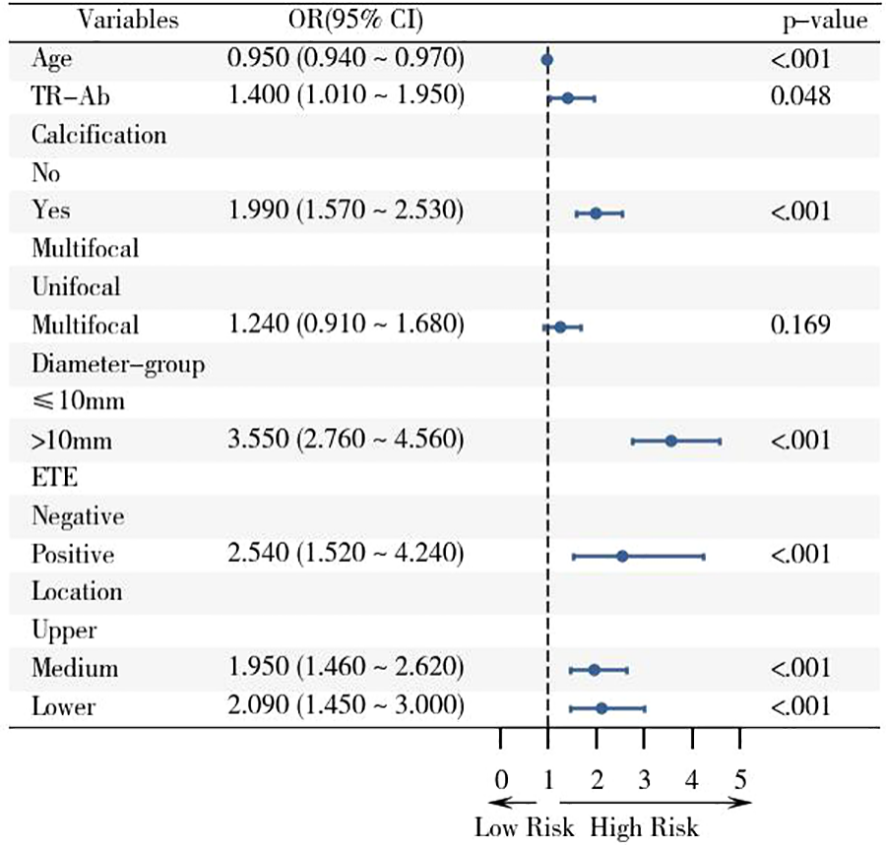
Forest plot of risk factors associated with CLNM in the training cohort based on logistic regression analysis with 95% confidence intervals. The screened variables were included in the logistic regression analysis, and the OR value and 95% CI of the multivariate analysis were displayed with the aid of the forest plot.

The results of multivariate regression analysis suggested that the presence of calcification (OR: 1.99, 95% CI: 1.57–2.53), maximum tumor diameter exceeding 10 mm (OR: 3.55, 95% CI: 2.76–4.56), ETE (OR: 2.54, 95% CI: 1.52–4.24), tumor localization in the mid-thyroid region (OR: 1.95, 95% CI: 1.46–2.62) and lower pole (OR: 2.09, 95% CI: 1.45–3.00), younger patient age, and high serum TR-Ab levels (p < 0.01) were identified as independent risk factors for CLNM in patients with PTC. Detailed results are presented in **Table 6**, and the forest plots of the results of the multivariate analysis are shown in **Figure 2**.

**Table 6:** Univariate and multivariate logistic regression analysis of training cohort.

Variables	Univariate		Multivariate	
	P	OR (95%CI)	P	OR (95%CI)
Age	**<.001**	0.95 (0.95 ~ 0.96)	**<.001**	0.95 (0.94 ~ 0.97)
TR-Ab(IUml)	**<.001**	1.59 (1.21 ~ 2.08)	**0.048**	1.40 (1.01 ~ 1.95)
Sex				
female		1.00 (Reference)		1.00 (Reference)
male	**0.019**	1.27 (1.04 ~ 1.55)	0.759	1.04 (0.81 ~ 1.35)
Calcification				
No		1.00 (Reference)		1.00 (Reference)
Yes	**<.001**	3.12 (2.58 ~ 3.77)	**<.001**	1.99 (1.57 ~ 2.53)
Multifocal				
Unifocal		1.00 (Reference)		1.00 (Reference)
Multifocal	**<.001**	1.62 (1.28 ~ 2.05)	0.169	1.24 (0.91 ~ 1.68)
Diameter-group,				
≤10mm		1.00 (Reference)		1.00 (Reference)
>10mm	**<.001**	3.78 (3.10 ~ 4.60)	**<.001**	3.55 (2.76 ~ 4.56)
ETE				
Negative		1.00 (Reference)		1.00 (Reference)
Positive	**<.001**	2.85 (1.88 ~ 4.30)	**<.001**	2.54 (1.52 ~ 4.24)
Location				
Upper		1.00 (Reference)		1.00 (Reference)
Medium	**<.001**	1.64 (1.30 ~ 2.08)	**<.001**	1.95 (1.46 ~ 2.62)
Lower	**0.010**	1.47 (1.10 ~ 1.97)	**<.001**	2.09 (1.45 ~ 3.00)

The meaning of the bold values indicated that there were clear statistical differences in the variable in different central lymph node metastasis status groups of papillary thyroid carcinoma (p < 0.05).

### 3.4 Construction and evaluation of nomogram prediction model

**Figure 3:**
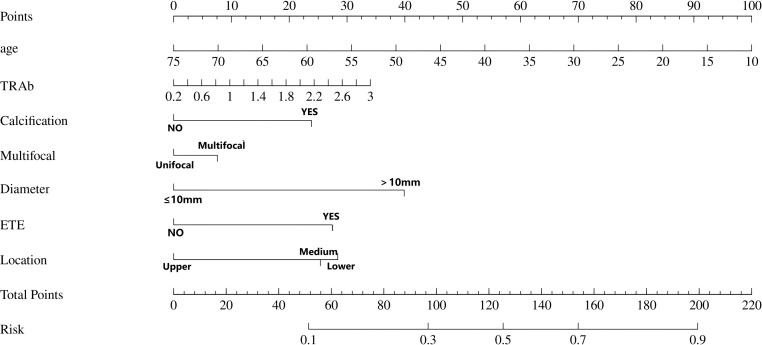
The Nomogram prediction model for the risk of central lymph node metastasis. In this predictive model, “Points” denote the weighted scores assigned to each risk factor (e.g., calcification, ETE) associated with CLNM based on their clinical status. For individual cases, clinicians calculate the “Total Points” by summing the individual scores corresponding to the patient’s specific clinical parameters within this nomogram model. This cumulative score is then projected onto the “Risk” axis to quantitatively estimate the probability of CLNM occurrence. ETE, extrathyroidal extension; location, Location of thyroid nodules (upper pole, middle pole, lower pole).

Utilizing the outcomes of logistic analyses, we constructed a Nomogram prediction model to predict the CLNM status of PTC patients by including the variables screened earlier (**Figure 3**). ROC, calibration curves, and DCA were used to verify the multidimensional effectiveness of the constructed Nomogram model.

**Figure 4:**
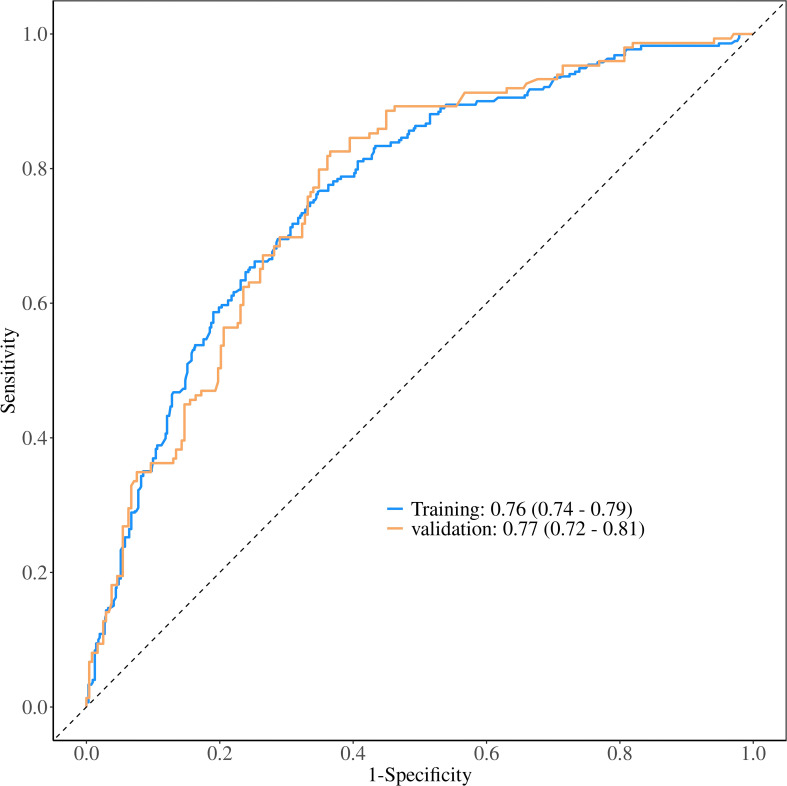
Comparative ROC curve analysis of the nomogram prediction model in training and validation cohorts.

**Figure 5:**
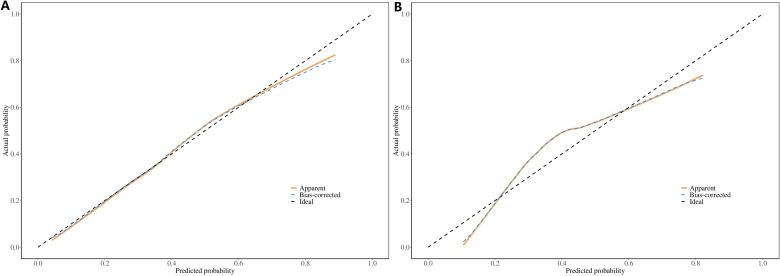
Calibration curves of the Nomogram prediction model: **(A)** Training Cohort (Left Panel), **(B)** Validation Cohort (Right Panel).

**Figure 6:**
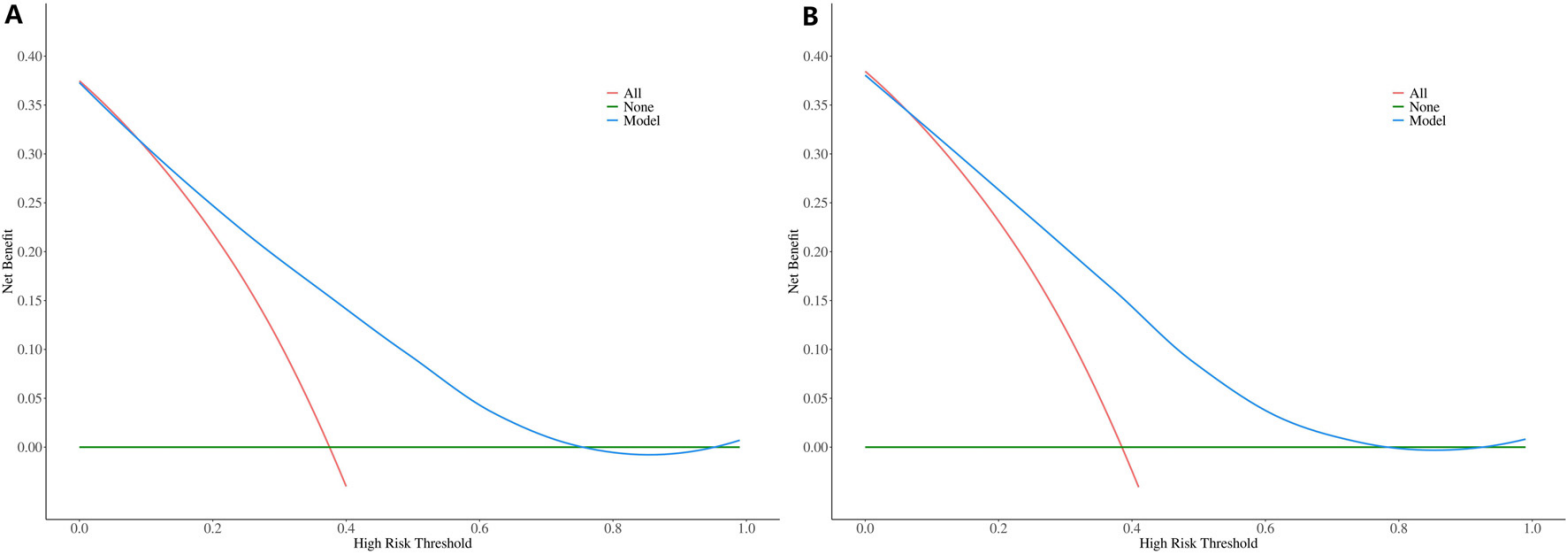
DCA curves of the Nomogram prediction model: **(A)** Training Cohort (Left Panel), **(B)** Validation Cohort (Right Panel).

The ROC constructed in the training cohort is shown in **Figure 4**, and the AUC was 0.76(95%CI: 0.74-0.79). To evaluate the generalizability and robustness of the model, The validation cohort, consisting of 487 patients, demonstrated robust discriminatory performance with an AUC of 0.77 (95% CI: 0.72-0.81). The consistency between the predicted probability of the model and the actual observed probability was evaluated by the calibration curve (**Figure 5**). The results showed that the predicted risk and the true risk were distributed along the 45° diagonal for both the training and validation cohorts. and the Hosmer-Lemeshow (HL) test was used to evaluate the fit between the observed values and the predicted values of the model, and the p value of the HL test was 0.99, indicating that there was no significant difference between the predicted values of the model and the true values, and the model fit was good. In addition, the mean absolute error (MAE) of the calibration curve was 0.07 by 1000 Bootstrap resampling, which further verified the high prediction accuracy and calibration consistency of the model. For robustness validation, DCA was performed to assess the clinical applicability of the nomogram, In the DCA, the clinical benefit curves of prophylactic central lymph node dissection for all patients and those without central lymph node dissection were delineated. Subsequently, the clinical benefit curves of prophylactic central lymph node dissection for PTC patients with different risks of lymph node metastasis, according to this prediction model, were compared with the aforementioned curves. The clinical value of the model was evaluated. The results confirmed that, except for a small range of low threshold probabilities, if the risk of CLNM in a particular PTC patient lies between the DCA curve and the other two control curves, the clinical benefit of prophylactic central lymph node dissection for this selected PTC patient is higher (**Figure 6**). Moreover, the clinical benefit interval of the Nomogram model covered most clinical decision-making scenarios, suggesting its applicability in different risk stratification.

## 4 Discussion

Thyroid cancer is the most common malignancy of the endocrine system, with PTC representing the most frequently occurring histological subtype. Surgical intervention constitutes the primary therapeutic modality for PTC, largely due to the favorable survival prognosis and the absence of specific postoperative adjuvant therapies (10), iodine-131 therapy is predominantly reserved for PTC patients exhibiting distant metastases (11). Consequently, achieving complete resection is imperative in the management of PTC. Although guidelines pertaining to the surgical approach for primary PTC are continuously evolving, the management of regional lymph nodes remains inconsistent (12),with PLND currently being the main recommendation. While PLND facilitates comprehensive lymph node clearance, it concurrently elevates the risk of postoperative complications, including recurrent laryngeal nerve injury and transient hypocalcemia (13). Prior research has documented varying incidences of CLNM following PLND (14). Consequently, we propose that evaluating the likelihood of CLNM through preoperative assessments and intraoperative observations to identify high-risk PTC patients for PLND represents a viable clinical strategy. This approach seeks to enhance surgical precision and reduce postoperative complications.

In this study, we utilized logistic regression analyses to identify independent variables included in the study after removing the high collinearity variables. Univariate analysis indicated no statistical differences in weight, height, and BMI between different CLNM status groups.

Some previous studies have suggested that lymph node metastasis in PTC patients has a significant gender preference, and male patients are more likely to have CLNM and lateral lymph node metastasis(LLNM) (15, 16), and suggest that for male patients, Physical examination and imaging evaluation of cervical lymph node status should be emphasized before operation (17). The possible mechanisms include the high expression of androgen receptor in male PTC tissues and the promotion of tumor cell invasion through MAPK/ERK pathway. The infiltration abundance of regulatory T cells (Tregs, Foxp3+) in the tumor microenvironment of male patients is higher, indicating stronger immunosuppression, and male patients tend to have M2 macrophage polarization. All the above mechanisms suggest that the tumor of male PTC patients is more aggressive. However, some studies believe that estrogen is a strong stimulator of malignant thyroid nodules, and female PTC patients are often accompanied by multifocal lesions and a higher rate of lymph node metastasis (18). However, in this study, males do not exhibit a higher likelihood of CLNM compared to females. While univariate analysis and clinical experience suggest that males under 40 show a high risk of CLNM, we attempted to include gender as a variable in our nomogram model to more fully explore the relationship between gender and the risk of central lymph node metastasis in PTC patients. However, it did not show robust predictive significance. Therefore, the influence of gender on CLNM in PTC patients still needs to be further studied in a larger patient cohort.

### 4.1 Age

Some studies have verified that age is a significant predictive factor of CLNM and the rate of CLNM was higher in younger patients (19), our research yielded similar result. Reviewing previous studies, we categorized patients based on age <40 (20) and age <45 (21, 22). The results revealed that PTC patients younger than 40 had a higher incidence of CLNM (51.45% vs. 46.97%) compared to those categorized by age <45. Including different age group criteria in the analysis, we found that using <40 years as a cutoff displayed a more robust predictive value (OR: 1.74, 95% CI: 1.33–2.27) compared to age <45 (OR: 1.60, 95% CI: 1.23–2.09). These results confirm that younger PTC patients are more likely to experience CLNM, with age 40 potentially being a more accurate cutoff than age 45.

### 4.2 Primary tumor characteristics

Ultrasound features serve as the principal method for evaluating the characteristics of thyroid tumors and the condition of regional lymph nodes (23). In this study, we examined the influence of ultrasound features on CLNM in patients with PTC. Univariate analysis demonstrated associations between CLNM and factors such as tumor location, calcification, the number of tumor foci, and ETE. Further multivariate analysis indicated that tumor situated in the middle or lower poles, calcification, multifocality, and ETE emerged as significant independent predictor of CLNM in PTC patients.

In contrast to tumors located in the upper pole, PTC patients with tumors in the middle and lower poles exhibited a higher rate of CLNM (24) (upper pole vs. middle pole OR: 1.64, 95% CI: 1.33–2.02; upper pole vs. lower pole OR: 1.59, 95% CI: 1.09–1.83). Calcification is a common characteristic of PTC, and before formulating the strategy of clinical data collection, we defined granular and microcalcifications as pathological calcifications, while the large coarse calcifications with clear edges were classified as physiological calcifications (25). Multivariate analysis demonstrated that PTC patients with pathological calcifications demonstrated an elevated probability of occurrence CLNM compared to those without calcifications or with physiological calcifications (OR: 3.11, 95% CI: 2.62–3.69). This is consistent with the previous results regarding calcification and high TNM stage PTC (26).

Current research suggests that extrathyroidal invasion increases the rate of CLNM (27, 28). Our study compared the relationship between CLNM and whether PTC tumor foci invaded the thyroid capsule. The results confirmed that extrathyroidal extension is associated with a higher likelihood of CLNM (OR: 3.45, 95% CI: 2.38–5.00). Additionally, we examined the influence of the relationship between intraoperative tumor foci and the thyroid capsule regarding regional lymph node metastasis. The findings indicate that both simple invasion of the thyroid capsule (OR: 1.59, 95% CI: 1.27–1.99) and tumor breach the thyroid capsule (OR: 2.32, 95% CI: 1.74–3.09) increase the likelihood of regional lymph node metastasis. Moreover, tumor breach the thyroid capsule also heightens the CLNM rate compared to simple capsule invasion (OR: 1.46, 95% CI: 1.01–2.28) (29).

Tumor size has been demonstrated to be relevant to CLNM (30), and larger tumors generally exhibiting more aggressive behavior (31). In the TNM staging of PTC, The diameter of the tumor less than or equal to 2 centimeters is classified as T1. However, with advancements in ultrasound detection accuracy and the growing frequency of routine check-ups, the detectable rate of small thyroid tumors has increased, thereby reducing the clinical significance of the traditional staging standard. The T1a/T1b classification has demonstrated greater precision in guiding the clinical management PTC (32).In our research, we did not observe significant differences between the tumor sizes measured by ultrasound with the actual size of pathological samples obtained after surgical resection, suggesting that the tumor sizes measured by ultrasound can be reliably utilized in predictive analyses. Consistent with numerous studies, we categorized primary tumors according to T1a staging. Our results indicated that PTC patients with tumors larger than 10 mm experience a heightened risk of CLNM compared to those classified as T1a. However, some researchers propose that a cutoff of 7 mm may offer a more precise threshold for assessing this risk (33). In our study, employing 7 mm as a grouping criterion did not show any significant clinical advantage in regression analyses or enhance the predictive capability of the model.

### 4.3 Tumor multifocality

Previous studies have revealed that multifocality increases the possibility of CLNM (34). In our study, patients with multifocal PTC exhibited a higher rate of CLNM. We define multifocality as the presence of two or more tumor foci and further categorize it into two foci and three or more foci. The comparison revealed that patients with two tumor foci had a higher CLNM rate (OR: 1.61, 95% CI: 1.19–2.18) compared to unifocal PTC. However, patients with ≥3 tumor foci did not demonstrate a significantly higher lymph node metastasis rate (OR: 0.73, 95% CI: 0.78–1.73), possibly due to the small sample size of this group (165/2,435, 6.78%). In our predictive model, we included the maximum tumor diameter as a variable, using the largest tumor focus for multifocal cases. To further elucidate the relationship between number of tumor foci and CLNM, we compared the maximum diameters among different groups. The results indicated no statistically significant differences in maximum tumor diameters across the groups (unifocal vs. bifocal: 10.04 vs. 9.78, p=0.735; unifocal vs. ≥3 foci: 10.04 vs. 11.35, p=0.155; bifocal vs. ≥3 foci: 9.78 vs. 11.35, p=0.118). Overall, although multifocal lesions demonstrated a higher rate of CLNM compared with unifocal lesions, further research involving larger cohorts is essential to establish whether three or more foci significantly elevate the risk of CLNM.

### 4.4 Thyroid function

Previous studies have suggested that serum thyroid hormone levels do not have a clear correlation with the progression of PTC or CLNM (35). However, the relationship between thyroid-related autoantibodies and PTC or CLNM remains unclear (36). Some research indicates that PTC patients with concurrent chronic lymphocytic thyroiditis are more likely to present with multifocal, bilateral thyroid lesions and lymph node metastasis (37). Our findings did not show significant differences in serum thyroglobulin (Tg) levels and thyroglobulin antibody (Tg-Ab) levels between different CLNM status groups (p>0.05). Notably, the CLNM group had higher thyroid receptor antibody levels(TR-Ab) (0.48 ± 0.43) compared to the CLNM(-) group (0.42 ± 0.28) (p=0.004). Through multivariate analysis, serum thyroid receptor antibody levels were identified as a significant risk factor for CLNM in patients with PTC (38). These findings offer valuable insights for preoperative decision-making.

In this study, we utilized demographic characteristics, ultrasound features, and thyroid function results to develop a nomogram prediction model of CLNM, and the Area Under the Curve (AUC) was 0.76. Previous studies utilizing CT or PET features reported AUCs of 0.730 and 0.75 for predicting CLNM in patients with PTC (39, 40), suggesting that our model exhibits enhanced differentiation capability for CLNM. Moreover, in comparison to those studies, the clinical features we selected are more readily accessible, providing greater socioeconomic relevance while preserving clinical predictive accuracy.

This study has certain limitations. First, although our model showed consistent predictive accuracy in the validation cohort, the model was derived from a single-center study and lacked external validation from other hospitals. which may limit the reliability and representativeness of the findings. The results of single-center studies are susceptible to institution-specific factors (e.g.,equipment models and practice norms), and only represent the disease characteristics and treatment patterns of a specific institution or region. The internal validity of the findings is high, but the external validity is low. In addition, our center is one of the top cancer treatment centers in China, The department of imaging diagnosis, pathology and head and neck surgery have more experience in the diagnosis and treatment of PTC patients compared with other medical centers, and the detection rate of different primary tumor characteristics is higher. Differences in imaging and clinical testing capabilities across patient populations and medical centers may limit the generalizability of this model. Future studies should further expand the sample size and conduct studies in medical centers with different imaging and pathological diagnostic capabilities and surgical treatment levels to enhance the generalizability of the results. Additionally, because BRAF V600E mutation status was not routinely tested at our institution, corresponding clinical data could not be collected, and BRAF V600E mutation status could not be included in this study as an independent variable, which may limit the accuracy and generalizability of our conclusions. Subsequent studies should further explore the impact of BRAF V600E mutation status on the risk of CLNM in PTC patients, especially by integrating multi-omics data and clinical covariates.

In conclusion, our study revealed key risk factors for CLNM in PTC patients, including younger age, elevated serum TR-Ab levels, calcification, multifocality, extrathyroidal invasion, larger tumor size, and tumors located in the mid-thyroid region and lower pole of the thyroid. The nomogram model we developed provides valuable clinical insights for surgeons, enabling the formulation of more tailored surgical strategies for PTC patients, which may significantly improve patient outcomes.

## Data Availability

The original contributions presented in the study are included in the article/supplementary material. Further inquiries can be directed to the corresponding author.

## References

[1] Yuan L Ma L Xue H Song S. Relationship between the upregulation of Notch1 signaling and the clinical characteristics of patients with papillary thyroid carcinoma in East Asia: a systematic review and meta-analysis. Cancer Cell Int. (2019) 19:5. doi: 10.1186/s12935-018-0723-8 30622441 PMC6317185

[2] Lubitz CC Sadow PM Daniels GH Wirth LJ. Progress in treating advanced thyroid cancers in the era of targeted therapy. Thyroid. (2021) 31:1451–62. doi: 10.1089/thy.2020.0962 PMC8591086 33860688

[3] Wang F Zhao S Shen X Zhu G Liu R Viola D. BRAF V600E confers male sex disease-specific mortality risk in patients with papillary thyroid cancer. J Clin Oncol. (2018) 36:2787–95. doi: 10.1200/JCO.2018.78.5097 PMC6145834 30070937

[4] Wang Z Chang Q Zhang H Du G Li S Liu Y. A clinical predictive model of central lymph node metastases in papillary thyroid carcinoma. Front Endocrinol (Lausanne). (2022) 13:856278. doi: 10.3389/fendo.2022.856278 35784530 PMC9243300

[5] Zhao Y Shi W Dong F Wang X Lu C Liu C. Risk prediction for central lymph node metastasis in isolated isthmic papillary thyroid carcinoma by nomogram: A retrospective study from 2010 to 2021. Front Endocrinol (Lausanne). (2022) 13:1098204. doi: 10.3389/fendo.2022.1098204 36733797 PMC9886574

[6] Dolcetti V Lori E Fresilli D Del Gaudio G Di Bella C Pacini P. US evaluation of topical hemostatic agents in post-thyroidectomy. Cancers (Basel). (2023) 15(9):2644. doi: 10.3390/cancers15092644 37174110 PMC10177612

[7] Buckle T Hensbergen AW van Willigen DM Bosse F Bauwens K Pelger RCM. Intraoperative visualization of nerves using a myelin protein-zero specific fluorescent tracer. EJNMMI Res. (2021) 11:50. doi: 10.1186/s13550-021-00792-9 34052912 PMC8164657

[8] Woo J Kwon H. Optimal surgical extent in patients with unilateral multifocal papillary thyroid carcinoma. Cancers (Basel). (2022) 14(2):432. doi: 10.3390/cancers14020432 35053595 PMC8773701

[9] Cheng P Xiang Y Chen E Zou Z Zhang X. Papillary thyroid microcarcinoma with synchronous asymptomatic advanced esophageal squamous cell carcinoma: A case report and review of the literature. Oncol Lett. (2015) 9:731–4. doi: 10.3892/ol.2014.2748 PMC4301557 25624899

[10] Jeong S Lee SG Kim H Lee G Park S Kim IK. Simultaneous expression of long non-coding RNA FAL1 and extracellular matrix protein 1 defines tumour behaviour in young patients with papillary thyroid cancer. Cancers (Basel). (2021) 13(13):3223. doi: 10.3390/cancers13133223 34203279 PMC8268647

[11] Du W Shi X Fang Q Zhang X Liu S. Feasibility of apatinib in radioiodine-refractory differentiated thyroid carcinoma. Front Endocrinol (Lausanne). (2022) 13:768028. doi: 10.3389/fendo.2022.768028 35282451 PMC8904562

[12] Adam MA Pura J Goffredo P Dinan MA Hyslop T Reed SD. Impact of extent of surgery on survival for papillary thyroid cancer patients younger than 45 years. J Clin Endocrinol Metab. (2015) 100:115–21. doi: 10.1210/jc.2014-3039 PMC5399499 25337927

[13] Robinson TJ Thomas S Dinan MA Roman S Sosa JA Hyslop T. How many lymph nodes are enough? Assessing the adequacy of lymph node yield for papillary thyroid cancer. J Clin Oncol. (2016) 34:3434–9. doi: 10.1200/JCO.2016.67.6437 PMC6366339 27528716

[14] Chen P Cai X Mu G Duan Y Jing C Yang Z. Supramolecular nanofibers co-loaded with dabrafenib and doxorubicin for targeted and synergistic therapy of differentiated thyroid carcinoma. Theranostics. (2023) 13:2140–53. doi: 10.7150/thno.82140 PMC10157742 37153748

[15] Ahn JE Lee JH Yi JS Shong YK Hong SJ Lee DH. Diagnostic accuracy of CT and ultrasonography for evaluating metastatic cervical lymph nodes in patients with thyroid cancer. World J Surg. (2008) 32:1552–8. doi: 10.1007/s00268-008-9588-7 18408961

[16] Tao L Zhou W Zhan W Li W Wang Y Fan J. Preoperative prediction of cervical lymph node metastasis in papillary thyroid carcinoma via conventional and contrast-enhanced ultrasound. J Ultrasound Med. (2020) 39:2071–80. doi: 10.1002/jum.v39.10 32352187

[17] Zhou SL Guo YP Zhang L Deng T Xu ZG Ding C. Predicting factors of central lymph node metastasis and BRAF(V600E) mutation in Chinese population with papillary thyroid carcinoma. World J Surg Oncol. (2021) 19:211. doi: 10.1186/s12957-021-02326-y 34256769 PMC8278623

[18] Kumar A Klinge CM Goldstein RE. Estradiol-induced proliferation of papillary and follicular thyroid cancer cells is mediated by estrogen receptors alpha and beta. Int J Oncol. (2010) 36:1067–80. doi: 10.3892/ijo_00000588 PMC11968770 20372779

[19] Sezer A Celik M Yilmaz Bulbul B Can N Tastekin E Ayturk S. Relationship between lymphovascular invasion and clinicopathological features of papillary thyroid carcinoma. Bosn J Basic Med Sci. (2017) 17:144–51. doi: 10.17305/bjbms.2017.1924 PMC5474108 28284178

[20] Xu SY Yao JJ Zhou W Chen L Zhan WW. Clinical characteristics and ultrasonographic features for predicting central lymph node metastasis in clinically node-negative papillary thyroid carcinoma without capsule invasion. Head Neck. (2019) 41:3984–91. doi: 10.1002/hed.v41.11 31463972

[21] Mao J Zhang Q Zhang H Zheng K Wang R Wang G. Risk factors for lymph node metastasis in papillary thyroid carcinoma: A systematic review and meta-analysis. Front Endocrinol (Lausanne). (2020) 11:265. doi: 10.3389/fendo.2020.00265 32477264 PMC7242632

[22] Yan B Hou Y Chen D He J Jiang Y. Risk factors for contralateral central lymph node metastasis in unilateral cN0 papillary thyroid carcinoma: A meta-analysis. Int J Surg. (2018) 59:90–8. doi: 10.1016/j.ijsu.2018.09.004 30342280

[23] Andrioli M Carzaniga C Persani L. Standardized ultrasound report for thyroid nodules: the endocrinologist’s viewpoint. Eur Thyroid J. (2013) 2:37–48. doi: 10.1159/000347144 24783037 PMC3821499

[24] So YK Kim MJ Kim S Son YI. Lateral lymph node metastasis in papillary thyroid carcinoma: A systematic review and meta-analysis for prevalence, risk factors, and location. Int J Surg. (2018) 50:94–103. doi: 10.1016/j.ijsu.2017.12.029 29329789

[25] Pyo JS Kang G Kim DH Park C Kim JH Sohn JH. The prognostic relevance of psammoma bodies and ultrasonographic intratumoral calcifications in papillary thyroid carcinoma. World J Surg. (2013) 37:2330–5. doi: 10.1007/s00268-013-2107-5 23716027

[26] Bai Y Zhou G Nakamura M Ozaki T Mori I Taniguchi E. Survival impact of psammoma body, stromal calcification, and bone formation in papillary thyroid carcinoma. Mod Pathol. (2009) 22:887–94. doi: 10.1038/modpathol.2009.38 19305382

[27] Zheng X Peng C Gao M Zhi J Hou X Zhao J. Risk factors for cervical lymph node metastasis in papillary thyroid microcarcinoma: a study of 1,587 patients. Cancer Biol Med. (2019) 16:121–30. doi: 10.20892/j.issn.2095-3941.2018.0125 PMC6528461 31119052

[28] Vasileiadis I Karakostas E Charitoudis G Stavrianaki A Kapetanakis S Kouraklis G. Papillary thyroid microcarcinoma: clinicopathological characteristics and implications for treatment in 276 patients. Eur J Clin Invest. (2012) 42:657–64. doi: 10.1111/j.1365-2362.2011.02633.x 22168782

[29] Kamaya A Tahvildari AM Patel BN Willmann JK Jeffrey RB Desser TS. Sonographic detection of extracapsular extension in papillary thyroid cancer. J Ultrasound Med. (2015) 34:2225–30. doi: 10.7863/ultra.15.02006 26518279

[30] Ma B Wang Y Yang S Ji Q. Predictive factors for central lymph node metastasis in patients with cN0 papillary thyroid carcinoma: A systematic review and meta-analysis. Int J Surg. (2016) 28:153–61. doi: 10.1016/j.ijsu.2016.02.093 26944586

[31] Chai W Ye F Zeng L Li Y Yang L. HMGB1-mediated autophagy regulates sodium/iodide symporter protein degradation in thyroid cancer cells. J Exp Clin Cancer Res. (2019) 38:325. doi: 10.1186/s13046-019-1328-3 31331356 PMC6647330

[32] Fei Y Wang B Yao X Wu J. Factors associated with occult lateral lymph node metastases in patients with clinically lymph node negative papillary thyroid carcinoma: a systematic review and meta-analysis. Front Endocrinol (Lausanne). (2024) 15:1353923. doi: 10.3389/fendo.2024.1353923 39493782 PMC11527613

[33] Heng Y Yang Z Cao P Cheng X Tao L. Lateral involvement in different sized papillary thyroid carcinomas patients with central lymph node metastasis: A multi-center analysis. J Clin Med. (2022) 11(17):4975. doi: 10.3390/jcm11174975 36078905 PMC9456507

[34] Wang Z Qu L Chen Q Zhou Y Duan H Li B. Deep learning-based multifeature integration robustly predicts central lymph node metastasis in papillary thyroid cancer. BMC Cancer. (2023) 23:128. doi: 10.1186/s12885-023-10598-8 36750791 PMC9906958

[35] Wang L Chen J Yuan X Wang J Sun L Jiang J. Lymph node metastasis of papillary thyroid carcinoma in the context of Hashimoto’s thyroiditis. BMC Endocr Disord. (2022) 22:12. doi: 10.1186/s12902-021-00923-2 34986823 PMC8734374

[36] Fiore E Latrofa F Vitti P. Iodine, thyroid autoimmunity and cancer. Eur Thyroid J. (2015) 4:26–35. doi: 10.1159/000371741 25960959 PMC4404933

[37] Liu Y Lv H Zhang S Shi B Sun Y. The impact of coexistent hashimoto’s thyroiditis on central compartment lymph node metastasis in papillary thyroid carcinoma. Front Endocrinol (Lausanne). (2021) 12:772071. doi: 10.3389/fendo.2021.772071 34867817 PMC8635140

[38] Kitahara C.M., K.R.F. D Jørgensen JOL Cronin-Fenton D Sørensen HT. Benign thyroid diseases and risk of thyroid cancer: A nationwide cohort study. J Clin Endocrinol Metab. (2018) 103:2216–24. doi: 10.1210/jc.2017-02599 PMC6276704 29590402

[39] Peng Y Zhang ZT Wang TT Wang Y Li CH Zuo MJ. Prediction of central lymph node metastasis in cN0 papillary thyroid carcinoma by CT radiomics. Acad Radiol. (2023) 30:1400–7. doi: 10.1016/j.acra.2022.09.002 36220726

[40] Kim BS Kim SJ Kim IJ Pak K Kim K. Factors associated with positive F-18 flurodeoxyglucose positron emission tomography before thyroidectomy in patients with papillary thyroid carcinoma. Thyroid. (2012) 22:725–9. doi: 10.1089/thy.2011.0031 PMC3387768 22524470

